# Generalized EIS Measurement Method in Li-Ion Batteries

**DOI:** 10.3390/s26113472

**Published:** 2026-05-31

**Authors:** Juan María Nogales, Israel Corbacho, Francisco Romero-Galán, Miguel Á. Domínguez, Juan M. Carrillo

**Affiliations:** Department of Electrical, Electronic and Automation Engineering, University of Extremadura, 06006 Badajoz, Spain; juanmaria@unex.es (J.M.N.); israelcc@unex.es (I.C.); franciscorg@unex.es (F.R.-G.); jmcarcal@unex.es (J.M.C.)

**Keywords:** electrical impedance spectroscopy, magnitude-ratio and phase-difference detection, shunt impedance, Li-ion battery cells, embedded sensor system, parasitic correction, printed circuit board (PCB), state of charge (SoC), discharge current

## Abstract

This work presents the realization of a compact and embedded impedance-based sensor system for the characterization of lithium-ion batteries by means of electrical impedance spectroscopy (EIS). The analog magnitude-ratio and phase-difference detection (MRPDD) method is implemented and extended through a generalized formulation that models the shunt element as a frequency-dependent impedance and compensates the parasitic contributions of the printed circuit board. This reformulation corrects magnitude and phase errors introduced by the measurement hardware without increasing the overall complexity. The prototype comprises two main functional blocks: current-mode excitation and voltage-mode measurement. The excitation stage uses an operational transconductance amplifier and a power MOSFET to generate a voltage-controlled current source, whereas the sinusoidal voltage signal is generated by means of a direct digital synthesizer. The measurement chain relies on differential acquisition using instrumentation amplifiers and analog magnitude/phase detection based on the AD8302 vector detector under microcontroller control. The proposed method has been first validated by simulations using both a linear *RC* equivalent model and an extended Randles-type battery-equivalent model, and then experimentally characterized using a linear *RC* equivalent model of the device under test. Measurements show that the generalized formulation recovers the ideal impedance response in the presence of parasitic effects, both in the shunt device and in the printed circuit board. In the experimental validation with the *RC* model, a magnitude error of 1.65% is obtained at 1 kHz, which is adopted as the upper frequency limit for battery characterization, even though operation up to 10 kHz is possible. Phase measurements revealed that the input capacitive coupling of the vector detector, conceived for operation in the RF range, requires an adaptation for appropriate operation in the intended frequency range. The prototype has been also applied to the characterization of a commercial lithium-ion 18650 cell, enabling the measurement of battery impedance and the analysis of its dependence on the state-of-charge and on the discharge current.

## 1. Introduction

Electrical impedance spectroscopy (EIS) has established itself as an essential technique for the dynamic analysis of lithium-ion (Li-ion) batteries, enabling the estimation of internal parameters related to the state of charge (SoC) and the state of health (SoH) [1,2,3]. However, conventional instrumentation solutions are generally based on network analyzers or dedicated EIS laboratory equipment, which are bulky and costly, thereby limiting their applicability in embedded or field environments [4,5].

Recent approaches aimed at the miniaturization of EIS have adopted simplified measurement schemes that prioritize linearity, reproducibility, and low power consumption [6,7,8,9]. In embedded implementations, impedance can be estimated using different strategies, including synchronous or lock-in detection, frequency-response analysis (FRA) techniques, FFT-based processing, and converter-based perturbation methods [10]. These approaches can provide high accuracy and flexibility, but they usually require synchronized acquisition, digital signal processing, or additional control circuitry, which may increase the hardware and computational complexity of compact sensor nodes. In converter-based EIS systems, additional control-oriented strategies may also be required to preserve bus-voltage stability during the injection of periodic excitation signals [11]. Among them, the analog magnitude-ratio and phase-difference detection (MRPDD) method stands out, as it can obtain the impedance magnitude and phase using a minimum number of analog channels [12,13].

**Figure 1:**
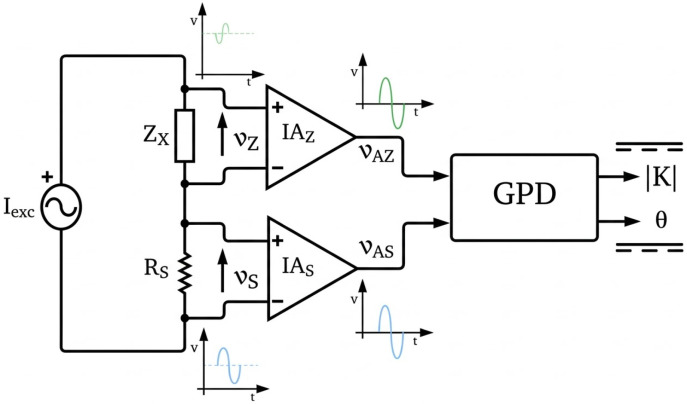
Simplified schematic of the MRPDD-based impedance extraction.

In addition, MRPDD provides a compact data representation, since the measurement can be reduced to two DC voltage levels corresponding to the outputs of a gain–phase detector (GPD), which provides the magnitude-ratio and phase-difference signals shown in Figure 1, avoiding the need to sample, store, and transmit full time-domain waveforms. This feature is especially attractive for compact embedded systems, since it reduces the requirements on data acquisition, storage, and digital signal processing, thereby limiting the need for high-performance signal-processing units. Although MRPDD has been previously proposed in impedance-measurement applications, to the best of the authors’ knowledge, it has not been explicitly applied to low-impedance Li-ion battery EIS with a generalized formulation that accounts for the non-ideal behavior of the shunt element and printed circuit board (PCB) parasitic effects. The classical formulation of this method assumes an ideal shunt resistor $R_{S}$ (see Figure 1) and neglects the parasitic effects associated with both the current-sensing element and the PCB. This simplification becomes especially critical in low-impedance measurements, where the parasitic behavior of low-value resistors may no longer be negligible at high frequency [14].

In EIS, impedance can be measured using either the potentiostatic approach (voltage stimulus, current sensing) or the galvanostatic method (current stimulus, voltage sensing). While the potentiostatic solution can typically reach higher stimulus frequencies, the galvanostatic method is often preferred in electrical applications because it provides a controlled excitation and avoids unintended large currents; moreover, potentiostatic setups usually require additional current-sensing circuitry [15]. For low-impedance energy storage systems, galvanostatic EIS is particularly convenient, since a controlled excitation current can be established and the resulting small voltage response can be measured with good sensitivity [15].

In this context, this work adopts a galvanostatic approach and proposes an embedded impedance spectroscopy system organized into an excitation stage and a measurement section for voltage acquisition and impedance extraction. The system implements a generalized version of the MRPDD method by explicitly modeling the shunt element as a complex, frequency-dependent impedance $Z_{S}\left(f\right)$. Furthermore, the method compensates the differential parasitic contributions of the PCB layout in the complex domain. Although the relevance of the frequency-dependent shunt impedance has already been recognized in conventional galvanostatic EIS setups [14], the proposed formulation incorporates this effect into the MRPDD framework and extends the impedance reconstruction by including the PCB parasitic contribution. This extension is critical for accurate very-low-impedance measurements, on the order of a few tenths of mΩ, while preserving a compact analog front-end and avoiding the need for intensive digital waveform processing.

To better position the proposed generalized MRPDD approach with respect to existing EIS implementations for Li-ion cells, Table 1 summarizes representative approaches from the literature and compares them with the system developed in this work. The comparison highlights that the proposed contribution is not limited to the implementation of a compact EIS prototype, but also includes a generalized impedance-reconstruction formulation that accounts for the non-ideal behavior of the shunt element and PCB parasitic effects within an analog MRPDD-based measurement chain. The remainder of the manuscript is organized as follows. Section 2 establishes the measurement principle and develops the generalized impedance measurement method. Section 3 then presents the architecture of the proposed EIS system. Section 4 examines the validity of the generalized method through system-level modeling and simulation. Section 5 reports the experimental assessment of the prototype, including results obtained with both an *RC* model and a Li-ion 18650 cell. Finally, Section 6 summarizes the main conclusions of this work.

## 2. Measurement Principle and Generalized Method

The developed system is based on the MRPDD method described in [12,13], which enables the determination of the complex impedance of a device under test (DUT), denoted as $Z_{X}$, by analog comparison of two sinusoidal signals: the voltage drop across the shunt element and the DUT. In the conventional configuration, a small-amplitude sinusoidal excitation current $I_{\mathrm{exc}}\left(t\right)$ is applied to the DUT, while the voltages $v_{Z}$ and $v_{S}$ across $Z_{X}$ and the shunt resistor $R_{S}$, respectively, are measured simultaneously. The instrumentation amplifiers, $IA_{Z}$ and $IA_{S}$, provide the amplified versions $v_{AZ}=A_{Z}\;v_{Z}$ and $v_{AS}=A_{S}\;v_{S}$, where $A_{Z}$ and $A_{S}$ denote the corresponding gains. As shown in Figure 1, a GPD processes $v_{AZ}$ and $v_{AS}$ and yields the magnitude-ratio |K| and the phase-difference θ between both signals.

In the following derivation, the sinusoidal voltages and currents are treated as complex phasors, although the same notation is retained for compactness. Under ideal conditions, the shunt element is assumed to be a purely resistive component $R_{S}$. Therefore, the voltage drops across the DUT and the shunt resistor can be written as

(1) $$v_{Z}=I_{\mathrm{exc}}\;Z_{X},\;v_{S}=I_{\mathrm{exc}}\;R_{S}.$$

Dividing both expressions, the DUT impedance is obtained from the voltage ratio as

(2) $$Z_{X}=R_{S}\;\frac{v_{Z}}{v_{S}}.$$

The amplified voltages can be expressed in phasor form as

(3) $$v_{AZ}=|v_{AZ}|∠\theta_{AZ},\;v_{AS}=\left|v_{AS}\right|∠\theta_{AS}.$$

Therefore, the complex ratio processed by the GPD is

(4) $$K=\frac{v_{AZ}}{v_{AS}}=\frac{|v_{AZ}|}{|v_{AS}|}∠\left(\theta_{AZ}-\theta_{AS}\right)=\left|K\right|∠\theta ,$$

with

(5) $$\left\{\begin{array}{c} \left|K\right|=\frac{|v_{AZ}|}{|v_{AS}|}\\ \theta =\theta_{AZ}-\theta_{AS} \end{array}\right.$$

where |K| is the magnitude-ratio of $v_{AZ}$ and $v_{AS}$, and θ is their phase-difference, with $\theta_{AZ}$ and $\theta_{AS}$ denoting the phases of $v_{AZ}$ and $v_{AS}$, respectively.

Combining (2) and (4), the conventional MRPDD expression becomes

(6) $$Z_{X}=R_{S}\;\frac{A_{S}}{A_{Z}}\;K=R_{S}\;\frac{A_{S}}{A_{Z}}\;\left|K\right|∠\theta .$$

Although the shunt element is physically implemented and labeled in the schematics as the resistor $R_{S}$, in the generalized formulation it is treated as a frequency-dependent complex impedance $Z_{S}$ to account for its non-ideal behavior.

To improve accuracy, the proposed generalized version of the MRPDD method replaces the ideal shunt resistor, $R_{S}$, by its complex, frequency-dependent shunt impedance $Z_{S}$, while also introducing a correction for the parasitic impedance of the system, represented by $Z_{par}$. In this case, the shunt impedance is expressed as

(7) $$Z_{S}=\left|Z_{S}\right|∠\theta_{S},$$

and the corresponding voltage drops are

(8) $$v_{Z}=I_{\mathrm{exc}}\;Z,\;v_{S}=I_{\mathrm{exc}}\;Z_{S},$$

where *Z* denotes the impedance reconstructed from the measured voltage ratio before subtracting the PCB parasitic contribution. Dividing both expressions in (8) gives

(9) $$Z=Z_{S}\;\frac{v_{Z}}{v_{S}}.$$

Using the complex ratio measured by the GPD, the generalized expression of the measured impedance becomes

(10) $$Z=Z_{S}\;\frac{A_{S}}{A_{Z}}\;K.$$

Since $Z_{S}=\left|Z_{S}\right|∠\theta_{S}$ and K=|K|∠θ, the product in (10) is performed in polar form, so that the magnitudes are multiplied and the phases are added. Therefore,

(11) $$Z=|Z_{S}|\cdot \frac{A_{S}}{A_{Z}}\cdot \left|K\right|∠\left[\theta +\theta_{S}\right],$$

where $|Z_{S}|$ and $\theta_{S}$ are the magnitude and phase of $Z_{S}$, respectively. Finally, the effective impedance of the DUT is obtained by subtracting the complex contribution of the parasitics:

(12) $$Z_{X}=Z-Z_{par}.$$

Accordingly, the DUT impedance can be expressed as $Z_{X}=\left|Z_{X}\right|∠\theta_{X}$, where $|Z_{X}|$ is the impedance magnitude and $\theta_{X}$ is its phase. This formulation simultaneously corrects the effects of the non-ideal shunt impedance and PCB trace asymmetries, avoiding the use of additional analog channels or intensive digital processing. The validity of the generalized method is further demonstrated in Section 4 through simulation results and in Section 5.1 by experimental results.

## 3. System Architecture

**Figure 2:**
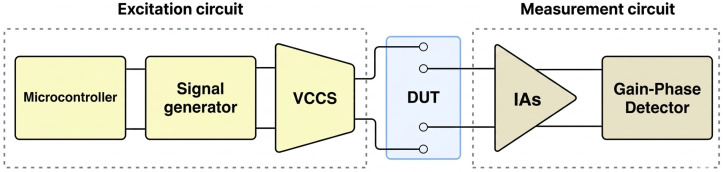
Block diagram of the proposed system.

The block diagram of the proposed EIS system, shown in Figure 2, includes the excitation and measurement sections previously described. The excitation block consists of the microcontroller, the signal generator, and a voltage-controlled current source (VCCS) that provides the excitation current applied to the DUT. The measurement block features differential voltage acquisition using instrumentation amplifiers (IAs) and a GPD that supplies the magnitude-ratio and phase-difference needed for MRPDD-based impedance extraction. Each block is designed to optimize stability, linearity, and reproducibility of the impedance measurement across a frequency range from 0.9 Hz to 1 kHz, even though operation out of this span is also possible and is reported in Section 4.

### 3.1. Generation of the Excitation Signal

**Figure 3:**
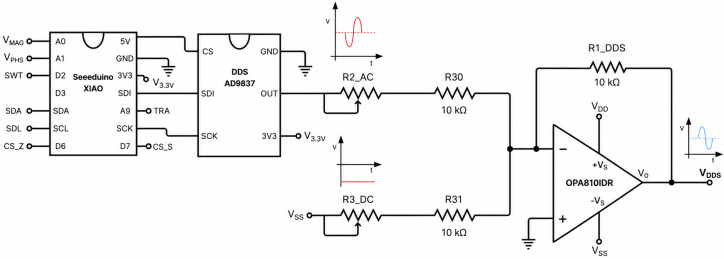
Generation and adjustment of the excitation signal using an inverting summing amplifier with OPA810IDR.

The excitation signal is generated by the AD9837 direct digital synthesizer (DDS), controlled via the serial peripheral interface (SPI) from the Seeeduino XIAO. As shown in Figure 3, the DDS output (600 mV_pp_, centered at a DC level of 1.65 V) is conditioned by an inverting summing amplifier based on the OPA810IDR, which allows independent adjustment of the AC and DC components, as shown in the next expression.

(13) $$V_{\mathrm{DDS}}=-\left(\frac{R_{1\mathrm{DDS}}}{R_{2\mathrm{AC}}+R_{30}}\;V_{\mathrm{AC}}+\frac{R_{1\mathrm{DDS}}}{R_{3\mathrm{DC}}+R_{31}}\;V_{\mathrm{DC}}\right).$$

The series resistors $R_{30}$ and $R_{31}$, chosen equal to the feedback resistor $R_{1\mathrm{DDS}}$, limit the gain of the overall configuration to 1 in magnitude (i.e., |gain|≤1) for any setting of the potentiometers $R_{2\mathrm{AC}}$ and $R_{3\mathrm{DC}}$. Therefore, the inverting summer always operates as an attenuator (never providing amplification), which prevents saturation and preserves linear operation in the intended frequency range.

### 3.2. Voltage-Controlled Current Source

**Figure 4:**
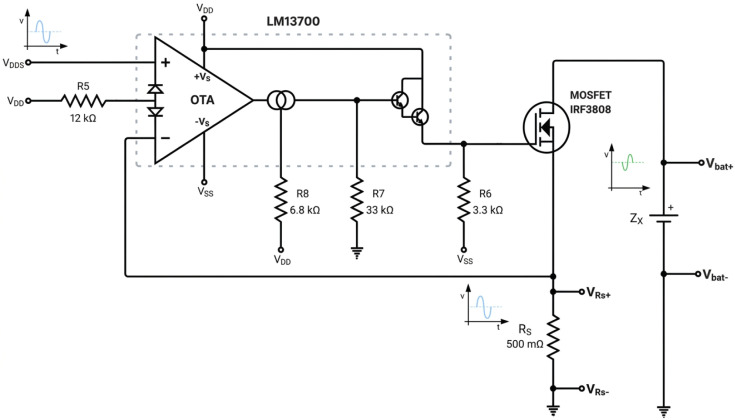
Current generation block: LM13700 OTA with Darlington buffer and IRF3808 MOSFET with $R_{S}$.

As shown in Figure 4, the signal $V_{\mathrm{DDS}}$ biases an LM13700 operational transconductance amplifier (OTA) configured as a VCCS, whose output (through its internal Darlington buffer) drives the gate of a power MOSFET, model IRF3808.

The resulting current flows through $Z_{X}$ and $R_{S}$, maintaining a high output impedance and a wide compliance voltage range. By virtue of the virtual short at the OTA input, $V_{\mathrm{DDS}}$ drops across $R_{S}$, separating the DC and AC components of the discharge current:

(14) $$I_{exc}\left(t\right)=\frac{V_{\mathrm{DC}}}{R_{S}}+\frac{V_{\mathrm{AC}}}{R_{S}}\;\sin\left(\omega t\right).$$

The sizing of auxiliary resistors $R_{6}$ to $R_{8}$, and the current limiting in the OTA output/linearizing diodes, stabilize the dynamics and prevent overdriving the MOSFET. In particular, since the OTA output is a current, $R_{7}$ provides the required current-to-voltage conversion at the Darlington-buffer input, establishing the drive voltage for the buffer stage, whereas $R_{6}$ converts the Darlington-buffer output current into the MOSFET gate-control voltage and provides a discharge path for the gate capacitance. Resistor $R_{8}$ sets the OTA amplifier bias current $I_{\mathrm{bias}}$ (and thus the transconductance $G_{m}$), enabling a controlled excitation-current amplitude. Finally, $R_{5}$ biases the LM13700 linearizing diodes and limits their current, improving large-signal linearity and avoiding excessive drive.

More generally, stability in EIS systems can be addressed at both the system-control and circuit levels. In converter-based online EIS implementations, an enhanced extended state observer has been proposed to mitigate bus-voltage fluctuations caused by periodic EIS perturbations in multi-stack electrochemical systems [11]. This approach is complementary to the present work: while observer-based control improves system-level robustness in power-converter EIS platforms, the proposed solution focuses on the hardware stability of a dedicated low-frequency battery-EIS front-end. Accordingly, the OTA-based VCCS is selected to improve current-source controllability and stability margin while maintaining low analog complexity.

**Figure 5:**
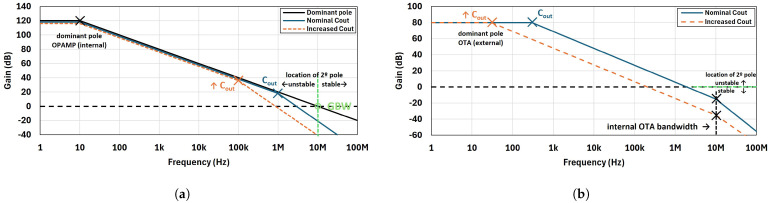
Stability comparisonbetween (**a**) a conventional operational amplifier and (**b**) an OTA, adapted from [21].

As reported in [20], an OTA-based Howland current source for bioelectrical impedance spectroscopy was shown to provide an approximately constant output current over 10 Hz–100 kHz while driving load impedances in the 1–10 kΩ range, supporting the choice of an OTA to improve current-source stability and controllability. In this work, the intended operating band (0.9 Hz–1 kHz) overlaps the range investigated in [20] over 10 Hz–1 kHz and extends it towards lower frequencies; therefore, the validity of the OTA-based excitation stage is experimentally assessed in our target range. Moreover, the OTA transconductance $G_{m}$ can be adjusted through the external bias current $I_{\mathrm{bias}}$ (set in this design by resistor $R_{8}$), providing a convenient knob to set the excitation-current amplitude and reduce sensitivity to load variations. A key difference between OTA and op-amp based architectures arises when analyzing the location of the stability-related poles as illustrated in Figure 5. In a conventional op-amp, the dominant pole is set internally and the second pole depends on the output resistance and the input capacitance of the power transistor ($C_{\mathrm{out}}$), which reduces the phase margin as $C_{\mathrm{out}}$ increases. In contrast, for an OTA the dominant pole is established at its output node, so that increasing $C_{\mathrm{out}}$ shifts it towards lower frequencies, improving phase stability as long as the gain-bandwidth product (GBW) remains at least one decade above the operating frequency [21].

### 3.3. Differential Acquisition

Two voltage drops are measured: across the impedance $Z_{X}$, denoted as $v_{Z}$, and across the shunt $R_{S}$, denoted as $v_{S}$. Both signals are acquired through identical differential conditioning stages based on the AD8429 instrumentation amplifier.

**Figure 6:**
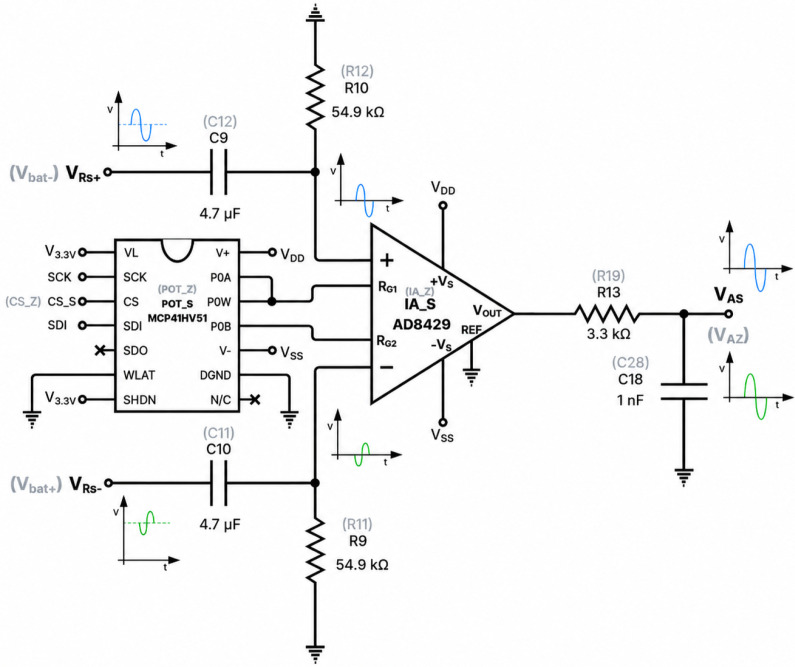
Differential acquisition scheme for the $v_{S}$ channel. The corresponding labels for the $v_{Z}$ channel are indicated in gray and in parentheses, since both acquisition paths are implemented with the same topology.

For the sake of compactness, Figure 6 shows the schematic corresponding to the $v_{S}$ channel, while the equivalent node names and component references of the $v_{Z}$ channel are indicated in gray and in parentheses. Each instrumentation amplifier is powered at ±7 V with terminal REF, used to set the DC level of the output voltage, tied to ground. AC coupling is used at the IAs’ inputs with $C_{9–10}=4.7\;\mu$F and $R_{9–10}=54.9\;$kΩ ($f_{c}\approx 0.6$ Hz) to block the DC component of the input signals and avoid saturation of the instrumentation amplifier. The gain of the AD8429 device is adjusted via an MCP41HV51-103E 10 kΩ digital potentiometer connected between its $R_{G}$ pins according to the expression:

(15) $$G=1+\frac{6\;k\Omega }{R_{G}}.$$

This 8-bit device provides 256 programmable wiper settings. For the 10 kΩ version, the nominal step resistance is $R_{AB}/255\approx 39.2\;\Omega$. Considering the resistor ladder and the typical wiper resistance, the implemented $R_{G}$ range extends approximately from 75Ω to 10,075Ω. According to (15), this corresponds to an instrumentation-amplifier gain range from G≈81 down to G≈1.6V/V. Although the AD8429 component can theoretically reach gains up to G=1000V/V, such high gain values are not required in the proposed measurement chain. In practice, the intended operating range is approximately G=2–15V/V, which provides sufficient flexibility to adapt the signal level while avoiding unnecessary amplification and saturation.

To preserve the correct relative phase relationship between $v_{Z}$ and $v_{S}$, the differential inputs of the instrumentation amplifier associated with $Z_{X}$ are connected with reversed polarity with respect to those of the $R_{S}$ channel.

### 3.4. Magnitude and Phase Detection

**Figure 7:**
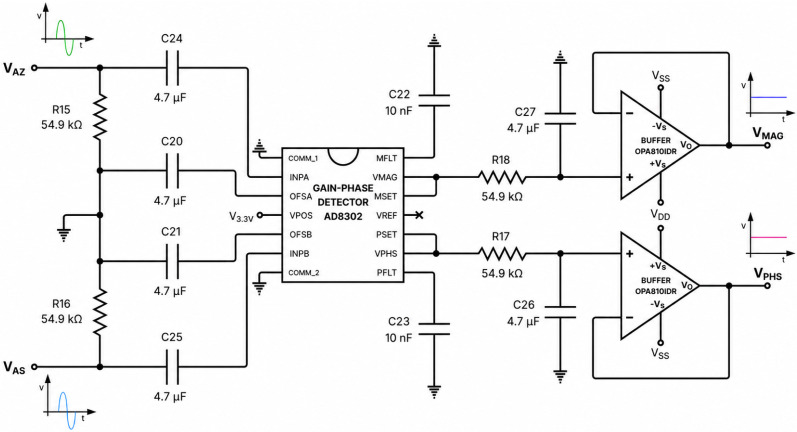
Magnitude and phase detection stage based on the AD8302 and output buffers.

The magnitude/phase detector is implemented with an AD8302-based GPD, as shown in Figure 7. Although the AD8302 is specified for RF operation, its magnitude-ratio and phase-difference outputs may be adapted for operation at lower frequencies, provided that the external AC-coupling network is properly redesigned.

In the reference configuration recommended in the datasheet [22], the 52.3Ω resistors are used for input-impedance adaptation, together with 1 nF coupling capacitors, which place the corresponding high-pass corner in the MHz range and therefore make this arrangement unsuitable for the EIS band considered in this work.

For the proposed system, the AC-coupling network was redesigned using 54.9kΩ resistors and 4.7μF capacitors. The selected resistor value preserves a near-unity gain in the adaptation stage, since the detector inputs are preceded by the 3.3kΩ low-pass-filter resistors located at the outputs of the instrumentation amplifiers. In this way, the input capacitive coupling of the vector detector, originally conceived for RF operation, is adapted for appropriate operation in the intended frequency range without relying solely on an excessive increase in capacitance.

This redesign should be understood as an adaptation of an RF-oriented detector to the low-frequency EIS range, rather than as a complete removal of all low-frequency phase limitations. Nevertheless, the phase output remains more sensitive than the magnitude output to residual phase shifts introduced by the coupling network, detector offsets, and buffering stages, especially near the lowest frequencies. For this reason, the PCB also provides test points at the outputs of the instrumentation amplifiers, allowing direct access to $v_{AZ}$ and $v_{AS}$ for phase validation. As discussed in Section 5, these test points were used in the present prototype to obtain the low-frequency phase values with an external precision oscilloscope.

The magnitude-ratio and phase-difference of the input signals can be extracted from the detector outputs as voltages $V_{\mathrm{MAG}}$ and $V_{\mathrm{PHS}}$, respectively, which follow these expressions [22]:

(16) $$\begin{array}{cc} V_{\mathrm{MAG}} & =600\;\mathrm{mV}/\mathrm{decade}\cdot \log_{10}\left(\frac{|V_{\mathrm{AZ}}|}{|V_{\mathrm{AS}}|}\right)+900\;\mathrm{mV}, \end{array}$$

(17) $$\begin{array}{cc} V_{\mathrm{PHS}} & =-10\;\mathrm{mV}/\deg\cdot \left(|\theta_{\mathrm{AZ}}-\theta_{\mathrm{AS}}|-90{}^{\circ}\right)+900\;\mathrm{mV}. \end{array}$$

Rearranging these expressions yields:

(18) $$\begin{array}{cc} \left|K\right| & =10^{\left(\frac{V_{\mathrm{MAG}}-900\;\mathrm{mV}}{600\;\mathrm{mV}}\right)}, \end{array}$$

(19) $$\begin{array}{cc} \theta & =\pm \left(\frac{900\;\mathrm{mV}-V_{\mathrm{PHS}}}{10\;\mathrm{mV}/\deg}+90{}^{\circ}\right). \end{array}$$

### 3.5. Control and Visualization

**Figure 8:**
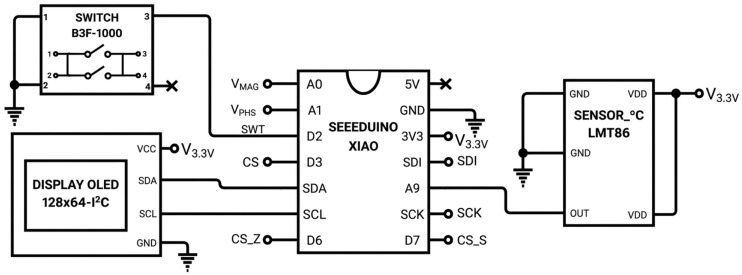
Control block (Seeeduino XIAO), visualization (OLED display), temperature sensor, and push button (B3F-1000).

The control and visualization block is implemented using a Seeeduino XIAO board, based on the ATSAMD21G18A-MU microcontroller from Microchip, featuring a 32-bit ARM Cortex-M0+ core. This device coordinates the different stages of the impedance measurement system. Figure 8 shows the general connection scheme with the peripherals.

The firmware handles the configuration of the excitation signal generator (AD9837) via SPI, defining the parameters of the frequency sweep, as well as the gain adjustment of the MCP41HV51 digital potentiometers associated with the instrumentation amplifiers to optimize the dynamic range. During operation, the internal ADC (12-bit, $V_{\mathrm{ref}}=3.3\;V$) performs the recording of $V_{\mathrm{MAG}}$ and $V_{\mathrm{PHS}}$ (from the GPD) together with the LMT86 temperature sensor output, followed by digital processing to compute the complex impedance $Z_{X}$ using the MRPDD equations. The results are visualized in real time on a 0.96” OLED I^2^C display, showing frequency, temperature, impedance magnitude and phase, and an estimated state of charge. User interaction is provided by a momentary push button (Omron B3F-1000), which triggers the start of the measurement sequence and whose completion is indicated on the display.

## 4. Validation of the Generalized Impedance Measurement Method

To validate the proposed generalized method for impedance measurement, simulations were carried out in PSpice considering equivalent battery models, as well as the parasitic effects associated with the reference resistor $R_{S}$ and the PCB itself. In addition to the linear *RC* model used as a controlled reference, an extended Randles-type equivalent model was included to assess the applicability of the proposed method to a more representative battery-equivalent impedance response.

### 4.1. System Modeling

**Figure 9:**
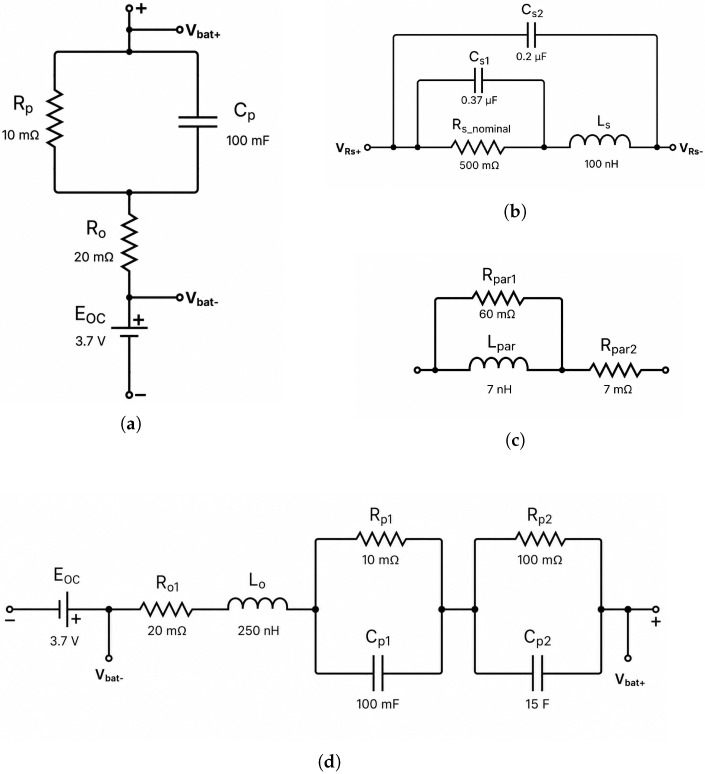
System modeling used for the validation of the generalized method: (**a**) equivalent *RC* model; (**b**) modeling of the non-ideal reference impedance $Z_{S}$; (**c**) modeling of the PCB parasitic impedance $Z_{par}$; (**d**) extended Randles-type model used as an additional battery-representative validation case.

As shown in Figure 9a, before applying the measurement methodology directly to a real Li-ion cell, an equivalent *RC* model is used to validate the operation of the prototype under controlled conditions. For this purpose, a Thévenin equivalent model is adopted due to its simplicity, its widespread use, and its ability to capture the dominant dynamic behavior of a cell with a compact set of parameters [23]. In its most basic form, the model assumes constant elements and consists of an ideal open-circuit voltage source $E_{\mathrm{OC}}$ in series with a resistor $R_{o}$, followed by a parallel branch $R_{p}\|C_{p}$.

More elaborated Thévenin-based models can incorporate additional elements to represent effects such as overload response and self-discharge under open-circuit conditions, and the parameters $E_{\mathrm{OC}}$, $R_{o}$, $R_{p}$, and $C_{p}$ are often made dependent on operating conditions (e.g., SoC, temperature, and charge/discharge current) to improve fidelity [23]. Here, $E_{\mathrm{OC}}=3.7\;V$ is implemented as a DC source that biases the equivalent network, while the small-signal impedance is determined by the *RC* elements in the frequency domain.

In this work, the simulation-based validation uses $R_{o}=20\;m\Omega$, $R_{p}=10\;m\Omega$, and $C_{p}=100\;\mathrm{mF}$, so that the characteristic time constant of the equivalent network falls within a frequency band comparable to that of the subsequent battery measurements, providing a linear DUT suitable for validating the measurement channel. With this value, the simulated Cole–Cole response of the *RC* model lies within the same 0.9Hz–10kHz validation range used for the extended Randles-type model. For the experimental validation of the *RC* model, however, the capacitor is implemented with a 4.7μF SMD ceramic device (X7S dielectric) due to its stable and nearly linear frequency response in the measurement band; in contrast, higher-capacitance technologies such as electrolytic capacitors (and some class-II ceramics) may exhibit pronounced frequency-dependent behavior and nonlinearity.

In simulations, the SMD resistors $R_{o}$ and $R_{p}$ are modeled as ideal components; nonetheless, similarly to the shunt element, practical low-value resistors may exhibit non-ideal parasitic effects, such as inductive behavior, whose influence becomes increasingly relevant as frequency rises. This issue has already been reported in galvanostatic EIS setups employing current-sensing shunts, where neglecting the frequency-dependent behavior of low-value resistors may introduce distortions in the measured response [14]. These effects are accounted for when interpreting the experimental results and further motivate the use of the generalized correction approach.

To extend the validation beyond the single-time-constant *RC* case, the extended Randles-type model shown in Figure 9d was also implemented. This model consists of an open-circuit voltage source $E_{\mathrm{OC}}$, a series ohmic resistance $R_{o1}=20\;m\Omega$, a small series inductance $L_{o}=250\;\mathrm{nH}$, and two parallel branches $R_{p1}\|C_{p1}$ and $R_{p2}\|C_{p2}$, with $R_{p1}=10\;m\Omega$, $C_{p1}=100\;\mathrm{mF}$, $R_{p2}=100\;m\Omega$, and $C_{p2}=15\;F$. The first polarization branch accounts for a charge-transfer/electric-double-layer contribution, whereas the second branch, with a much larger capacitance, introduces a slower diffusion-related trend in the low-frequency region. Although this model is still a linearized equivalent circuit, it was intentionally selected because it can be directly implemented in PSpice using lumped *R*, *L*, and *C* elements and co-simulated together with the complete excitation and measurement topology. Therefore, it provides a more representative validation scenario and can be co-simulated together with the complete system over the same 0.9Hz–10kHz validation range considered for the equivalent *RC* model. This simulated range includes the practical battery-characterization band later adopted in the experiments, namely 0.9Hz–1kHz.

The parasitic elements of the system were determined experimentally by means of impedance measurements on the PCB using an HP 4263B LCR meter. As shown in Figure 9b, the non-ideal shunt-impedance model ($Z_{S}$) includes the effects of series inductance ($L_{s}=100\;\mathrm{nH}$) and parasitic capacitances ($C_{s1}=0.37\;\mu F$, $C_{s2}=0.2\;\mu F$), together with a nominal resistance of $R_{S}=500\;m\Omega$. The physical reference element selected for $R_{S}$ was an RSPCS-series 500mΩ, ±0.5%, 3W, thin-film SMD current-sensing resistor in a 2512 package, with a temperature coefficient of ±100ppm/°C (RS stock number 241-4970, MPN RSPCS12DTERR500). The selection of this component is relevant because, in very-low-impedance measurements, the tolerance, temperature coefficient, and physical construction of the shunt resistor directly affect the accuracy and frequency dependence of the reference impedance. In particular, compact SMD thin-film implementations are preferred over bulkier wirewound or ceramic power resistors, which may introduce larger parasitic inductive effects. This modeling choice is consistent with previous studies showing that the current-sensing shunt must be treated as a frequency-dependent impedance, rather than as an ideal resistor, in order to avoid high-frequency measurement artifacts [14]. Similarly, as shown in Figure 9c, the PCB parasitic effects ($Z_{par}$) were modeled considering the track resistance ($R_{par1}=60\;m\Omega$), the trace inductance ($L_{par}=7\;\mathrm{nH}$), and the distributed resistance ($R_{par2}=7\;m\Omega$). The equivalent DUT models and the parasitic models were subsequently implemented in the simulation environment together with the measurement circuit, reproducing the real operating conditions of the physical system.

### 4.2. Simulation Results

**Figure 10:**
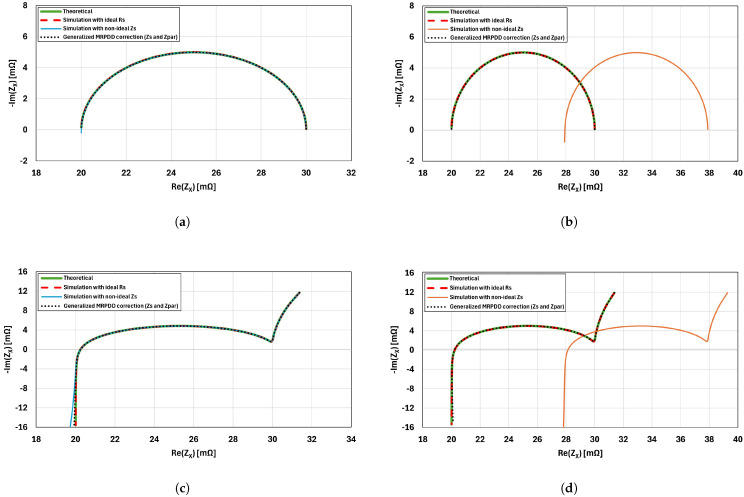
Simulation-based validation of the generalized MRPDD method for the equivalent *RC* model, upper row, and the extended Randles-type model, lower row. Subfigures (**a**,**c**) include only the non-ideal shunt impedance $Z_{S}$, corrected using (11), whereas subfigures (**b**,**d**) include both $Z_{S}$ and $Z_{par}$, corrected using (12). In all cases, the corrected response agrees with the theoretical impedance and with the simulation obtained using an ideal shunt resistor $R_{S}$.

To validate the generalized impedance-extraction method, Nyquist (Cole–Cole) plots were analyzed for both the equivalent *RC* model and the extended Randles-type model under different simulation conditions, as shown in Figure 10. In all cases, the simulated response obtained with the complete excitation and measurement topologies was compared with the theoretical impedance of the corresponding model and with the response obtained using an ideal shunt resistor $R_{S}=500\;m\Omega$. For consistency, both equivalent models were evaluated over the same simulated frequency range from 0.9Hz to 10kHz.

For the equivalent *RC* model, the theoretical curve, the simulation obtained with the ideal shunt resistor $R_{S}$, and the corrected response overlap almost exactly. The first validation case, presented in Figure 10a, evaluates only the effect of the non-ideal shunt impedance $Z_{S}$. In this case, the simulated response obtained with the non-ideal shunt model remains very close to the theoretical curve over most of the analyzed range, and only a slight deviation can be observed near the upper end of the simulated band, mainly in the inductive tail. Subsequently, the correction given by (11) is applied. The corrected response then overlaps again with both the theoretical curve and the simulation obtained with the ideal shunt resistor, as shown in Figure 10a, confirming that the generalized formulation compensates for the residual effect of $Z_{S}$ in this validation range. The second validation case, shown in Figure 10b, includes both the non-ideal shunt impedance $Z_{S}$ and the PCB parasitic contribution $Z_{par}$. Under these conditions, the uncorrected simulated response exhibits a much larger deviation with respect to the theoretical behavior. However, when the complete generalized correction defined by (12) is applied, the corrected impedance response again agrees closely with the theoretical result and with the simulation using the ideal resistor $R_{S}$, as can be seen in Figure 10b.

The same validation procedure was applied to the extended Randles-type model, as shown in Figure 10c,d. When only $Z_{S}$ is considered, the deviation of the uncorrected response with respect to the theoretical curve is still small over most of the 0.9Hz–10kHz validation range, indicating that the isolated effect of the frequency-dependent shunt parasitics remains limited in this case. Nevertheless, the corrected response overlaps with both the theoretical impedance and the simulation obtained using an ideal shunt resistor, confirming that the generalized formulation remains valid for this model as well. When both $Z_{S}$ and $Z_{par}$ are included, the uncorrected response exhibits a clearly visible deviation. After applying the complete correction given by (12), the corrected curve again matches the theoretical response and the simulation with ideal $R_{S}$.

Therefore, the results shown in Figure 10 confirm that the proposed generalized MRPDD method is not restricted to a simple single-time-constant *RC* benchmark, but also recovers the expected response of a more representative battery-equivalent model. In both cases, the theoretical impedance of the DUT can be accurately reconstructed even in the presence of parasitic effects associated with the shunt element and the PCB layout. In the analyzed validation range, the isolated contribution of $Z_{S}$ is relatively small, whereas the inclusion of $Z_{par}$ produces the most noticeable distortion in the uncorrected response, further supporting the need for the complete generalized correction.

## 5. Experimental Results

**Figure 11:**
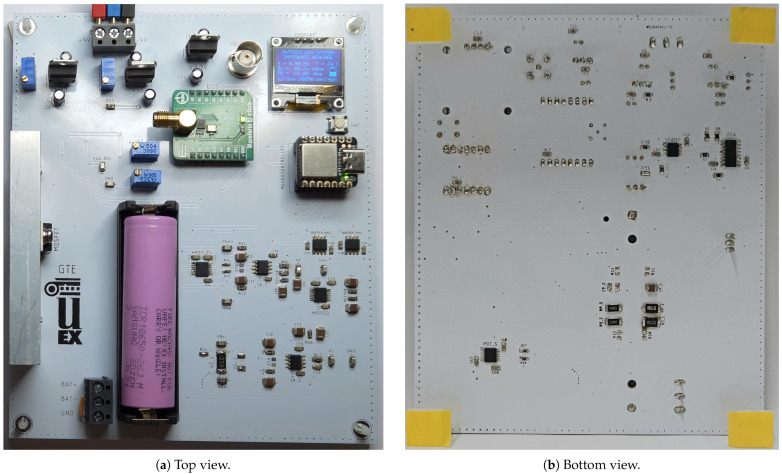
Photographs of the fabricated PCB prototype used for the experimental validation: (**a**) top view; (**b**) bottom view.

This section presents the experimental results obtained with the fabricated prototype. First, the system is validated under controlled conditions using the equivalent *RC* model introduced in Section 4. Then, the prototype is applied to the characterization of a commercial Li-ion 18650 cell under real operating conditions. Photographs of the PCB used in both experimental stages are shown in Figure 11. The prototype was implemented on a two-layer FR-4 PCB with a nominal thickness of 1.6mm, 35μm copper, solder mask on both sides, and top/bottom silkscreen. The layout includes common ground planes on both copper layers, interconnected by distributed stitching vias, while the power rails are routed using wider traces and local 100nF decoupling capacitors are placed close to the supply pins of the main integrated circuits. The high-current excitation path associated with the battery, MOSFET, and shunt element is routed using wider traces and kept separated, as far as possible, from the low-level differential sensing traces. The voltage drops across $Z_{S}$ and the DUT are acquired using a Kelvin-like sensing strategy, with separate current-carrying and voltage-sense traces. Since the shunt resistor is a two-terminal SMD component and the DUT voltage is sensed after a finite PCB path, the remaining layout contribution is experimentally characterized as $Z_{par}$ and explicitly included in the generalized correction method. To improve reproducibility, the PCB design and fabrication files, including the Gerber files and the 3D STEP model, are available in a public GitHub repository [24].

### 5.1. Prototype Validation with *RC* Model

For the initial validation of the prototype, the equivalent *RC* model shown in Figure 9a was used. It consists of a series resistor $R_{o}=20\;m\Omega$ and a parallel branch formed by $R_{p}=10\;m\Omega$ and $C_{p}=100\;\mathrm{mF}$. In the experimental implementation, the *RC* model was implemented on the bottom side of the same PCB used for the battery measurements (see Figure 11b), using an X7S ceramic capacitor of 4.7μF, since this component exhibits a sufficiently linear behavior within the analyzed frequency range. This model was used to compare theoretical, simulated, experimental, and corrected experimental results, together with reference measurements obtained using an HP 4263B LCR meter. The theoretical and simulated responses are represented up to 10MHz in order to show the overall trend of the Bode plots, whereas the experimental validation is limited to the range from 100Hz to 10kHz.

**Figure 12:**
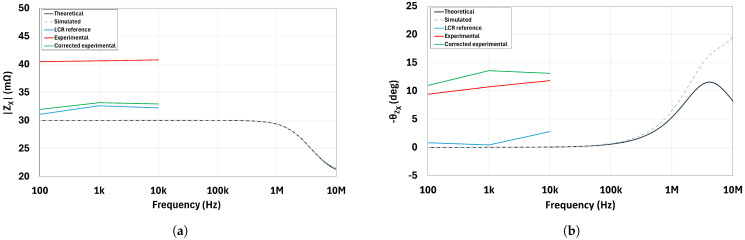
Bode plots of the impedance of the *RC* model: (**a**) magnitude response; (**b**) phase response. Theoretical and simulated results are shown up to 10MHz to illustrate the overall frequency trend of the implemented model, while the experimental, corrected experimental, and LCR-reference results are reported from 100Hz to 10kHz.

Figure 12 shows the Bode plots of the impedance magnitude and phase obtained for the *RC* model. The theoretical and simulated curves remain almost perfectly overlapped up to 10MHz. However, a small deviation of the simulated response with respect to the theoretical one can be observed in magnitude from 10MHz, while in phase this deviation becomes more significant from 1MHz.

Regarding the experimental validation, the observed behavior is consistent with the simulation-based validation presented in Section 4, where the generalized MRPDD method was shown to recover the intrinsic impedance in the presence of $Z_{S}$ and $Z_{par}$. In the experimental results, the corrected impedance magnitude also moves significantly closer to the LCR reference than the direct uncorrected measurement, thereby providing additional evidence that the proposed generalized method remains effective under real operating conditions. In particular, at 1kHz, the absolute deviation in impedance magnitude with respect to the LCR reference is approximately 0.54mΩ, corresponding to a relative error of 1.65%. Even when the frequency is increased to 10kHz, the deviation reaches only about 0.71mΩ, equivalent to a relative error of 2.21%. These results confirm that the generalized correction significantly improves the impedance-magnitude estimation despite the very low impedance levels under test.

It should be noted that the reported magnitude error of 1.65% at 1kHz represents the residual global error of the corrected measurement chain, rather than the isolated contribution of a single non-ideality. This residual deviation may include the combined effect of component tolerances, gain-setting uncertainty, detector non-idealities, soldering and interconnection effects, and parasitic elements not fully captured by the simplified models. Nevertheless, the simulation-based validation in Section 4 indicates that the PCB parasitic contribution $Z_{par}$ can introduce a more pronounced deviation than the non-ideal shunt impedance $Z_{S}$ in the considered frequency range. Therefore, the remaining error observed experimentally is expected to be mainly associated with residual PCB-related parasitic effects and practical implementation tolerances.

In contrast, the phase results obtained with the detector do not converge to the LCR reference, even after applying the generalized correction. This indicates that the main limitation in phase estimation is not associated with the $Z_{S}$ and $Z_{par}$ correction itself, but with the input capacitive coupling of the AD8302 vector detector, originally conceived for RF operation, which requires adaptation for proper use in the intended frequency range. However, when the phase is measured at the PCB test points specifically set up for this purpose, located at the outputs of the instrumentation amplifiers using a precision oscilloscope, the results are in good agreement with the reference. This confirms that the proposed excitation and measurement topologies are adequate, and that the limitation is specifically related to the phase detection stage of the AD8302. Consequently, in the subsequent battery measurements, the phase is obtained from the oscilloscope measurements at those test points.

Although the corrected magnitude error remains below 3% up to 10kHz in the *RC*-model validation, the different frequency ranges used in the experimental stages should be distinguished. The *RC* model was evaluated up to 10kHz, since this value represents a practical upper limit for battery EIS and remains within the useful frequency set of the HP 4263B LCR meter used as reference; the next available decade, 100kHz, is beyond the frequency range required for the intended battery characterization. The comparison with the LCR reference showed a slightly lower magnitude error at 1kHz than at 10kHz. Therefore, 1kHz was selected as a conservative upper limit for the battery measurements, providing sufficient impedance information while ensuring reduced measurement errors over the analyzed range. The lower limit of 0.9Hz was selected as a practical compromise to avoid excessively long frequency sweeps and to reduce the variation of SoC between the beginning and the end of each measurement under discharge conditions.

### 5.2. Battery Characterization

The battery measurements were carried out using the fabricated prototype. The top side of the PCB is shown in Figure 11a during the battery-impedance measurement, with the Li-ion 18650 cell mounted on the board during data acquisition. During the measurement process, the prototype applies a controlled discharge current to the battery under test and performs the frequency sweep. Through the measurement circuit, the impedance magnitude is obtained, while the phase-difference required for impedance reconstruction is measured with a precision oscilloscope at the PCB test points located at the outputs of the instrumentation amplifiers. Since these measurement nodes are still affected by the non-idealities associated with $Z_{S}$ and $Z_{par}$, both quantities are processed according to the generalized measurement method to reconstruct the battery impedance. The measured data are displayed in real time on the integrated OLED screen.

A commercial Samsung ICR18650–26J cell with a nominal capacity of 2600mAh was used. All measurements were carried out with a constant discharge current of 0.15C (approximately 0.4A), over a frequency range from 0.9Hz to 1kHz, and at room temperature, varying between 27 and 32°C, as monitored by the temperature sensor integrated on the PCB. Although the temperature was not actively controlled during these tests, the inclusion of this sensor is relevant for future studies addressing the correlation between impedance and temperature.

**Figure 13:**
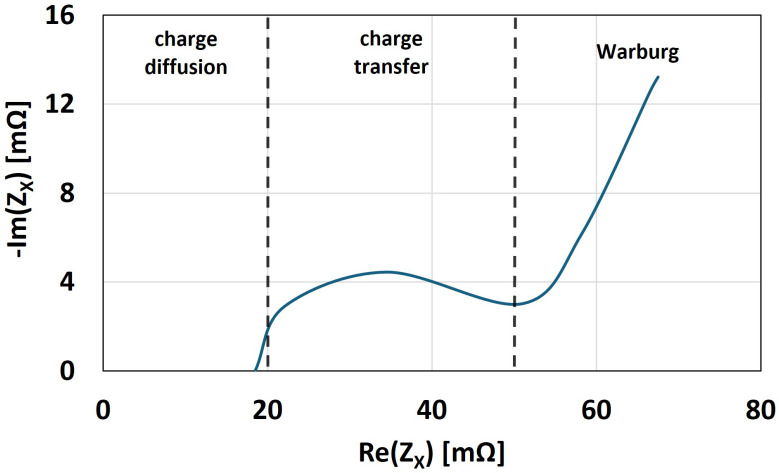
Cole–Cole diagram of the Li-ion 18650 battery at 100% SoC.

Figure 13 shows the experimental Cole–Cole plot corresponding to the battery at 100% SoC. The response exhibits a high-frequency intercept at low real-impedance values, followed by a depressed semicircular region associated with charge-transfer processes and a low-frequency tail commonly related to Warburg diffusion effects (these three characteristic regions are indicated in the figure by dashed lines). Overall, the measured response is consistent with the expected electrochemical behavior of a Li-ion cell.

It is worth noting that this experimental Cole–Cole response resembles the trend obtained with the extended Randles-type model introduced in Section 4, especially regarding the presence of a semicircular region and a low-frequency diffusion-related trend. This similarity further supports the use of the extended Randles-type model as an additional battery-representative validation case for the generalized method.

**Figure 14:**
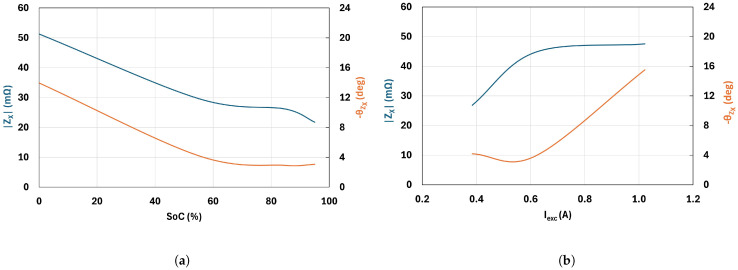
Low-frequency indicators extracted from the measured battery spectra: dependence of impedance magnitude and phase on (**a**) state of charge at 15Hz and (**b**) discharge current at 5Hz.

Subsequently, the dependence of the impedance on the SoC and on the discharge current was analyzed from the measured spectra. Instead of presenting a complete Cole–Cole diagram for each operating condition, Figure 14 summarizes the observed trends through representative low-frequency points, where the impedance variation is more pronounced than in the high-frequency region. In particular, the SoC dependence was evaluated at 15Hz, while the discharge-current dependence was evaluated at 5Hz. In Figure 14a, the magnitude of the impedance increases progressively as the SoC decreases, while the phase shifts to more negative values. In Figure 14b, both the impedance magnitude and the absolute phase angle increase with the applied discharge current, highlighting the impact of discharge overloading on the dynamic response of the electrochemical system and emphasizing the need for precise current control during battery characterization.

The obtained results demonstrate the practical applicability of the proposed prototype for the experimental characterization of real Li-ion cells and show impedance trends consistent with those reported in the literature for impedance spectroscopy measurements on Li-ion batteries. In particular, the increase in impedance magnitude at lower SoC values, shown in Figure 14a, and the dependence of the measured impedance on the discharge current, shown in Figure 14b, are both consistent with the expected behavior of Li-ion cells under different operating conditions [1,16].

### 5.3. Performance Summary of the Prototype

Beyond the individual Bode and Cole–Cole plots, it is useful to summarize the main performance figures of the embedded EIS prototype. Table 2 gathers the most relevant characteristics of the system, including the validated measurement range, excitation and acquisition strategy, and main implementation aspects. These values are derived from the simulation-based and experimental characterization performed on both the *RC* model and the commercial 18650 cell.

## 6. Conclusions

This work presents a generalized measurement method for impedance spectroscopy that explicitly corrects hardware non-idealities by incorporating both the real impedance of the reference element and the parasitic effects associated with the PCB. Simulation-based validation confirms that, even in the presence of non-ideal components and unwanted couplings, the method is able to accurately recover the complex impedance of the device under test, reproducing the ideal behavior of the equivalent model. This validation was first performed with a controlled *RC* equivalent model and then extended to a Randles-type equivalent model, showing that the proposed correction is not restricted to a single-time-constant benchmark but can also recover a more battery-representative impedance response.

After its validation, the method has been implemented in an embedded prototype that integrates a programmable sinusoidal excitation source, a current source based on an OTA and a MOSFET, differential acquisition with instrumentation amplifiers, and an analog magnitude-and-phase detector, all governed by a low-power microcontroller. Experimental tests show that the generalized method effectively corrects the deviations introduced by real-world electronics, enabling consistent results both on the equivalent *RC* model and on a commercial Li-ion cell. The main purpose of this work was therefore the proposal and validation of a generalized MRPDD formulation for low-impedance battery EIS, rather than the exhaustive electrochemical characterization of a specific cell chemistry.

Although the prototype demonstrated the feasibility of the proposed generalized MRPDD method, some integration aspects remain open. In particular, the impedance-magnitude readout is already embedded in the prototype, whereas the fully embedded low-frequency phase readout remains a pending integration challenge due to the RF-oriented input coupling of the AD8302 detector. In this prototype, the phase was therefore validated through dedicated PCB test points and an external precision oscilloscope. Future implementations will further investigate active coupling and capacitance-multiplication techniques, which are already under study, in order to adapt the detector input stage to low-frequency EIS without resorting to excessively large physical capacitors.

The results obtained on a 18650 cell exhibit trends that are consistent with the evolution of the state of charge and the discharge current, reinforcing the applicability of the proposed method—together with its embedded implementation—for integrated estimation of the SoC and, potentially, of the SoH in energy storage systems [25]. The experimental validation was carried out with a commercial ICR 18650 cell as a proof of concept. However, preliminary impedance measurements performed with other cylindrical cells available in the laboratory, including INR/NMC, NCR, and IFR chemistries in 18650 and 18500 formats, showed impedance ranges of the same order of magnitude as the ICR cell analyzed in this work. This is consistent with the diversity of commercial 18650 Li-ion chemistries reported in the literature, including IMR, INR, ICR, and IFR cells [26]. Therefore, the proposed excitation level and generalized reconstruction method are expected to be extendable to other Li-ion chemistries and formats, although systematic validation over a larger cell set is still required.

Future work will also address the influence of operating conditions. In the present experiments, temperature was monitored during the measurements and remained within a bounded range, but it was not actively controlled. Therefore, future tests should evaluate the temperature dependence of the measured impedance, as well as aging and cycling experiments aimed at correlating impedance evolution with SoH. Finally, the integration of EIS with complementary sensing techniques, such as ultrasonic sensing, may provide additional information for future multimodal SoC/SoH estimation strategies [27].

## Figures and Tables

**Table 1:** Comparison of representative EIS implementations for batteries and related MRPDD/GPD-based methods.

Work	Application	Method/Frequency Range	Key Distinction vs. This Work
**This work**	Embedded Li-ion battery EIS	Single-sine excitation; generalized MRPDD; explicit correction of $Z_{S}$ and $Z_{par}$; 0.9 Hz–1 kHz for battery measurements	Applies MRPDD to low-impedance Li-ion cells with explicit shunt/parasitic correction in an embedded prototype
[16]	Laboratory Li-ion battery EIS	Conventional galvanostatic single-sine EIS; 10 mHz–5 kHz	Classical battery EIS reference; not embedded and without simplified analog extraction
[7]	Embedded cell-level battery EIS/BMS	Single-sine perturbation with converter-based injection and digital processing; 25 mHz–7.66 kHz	Very relevant embedded benchmark, but with higher hardware and control complexity
[10]	Real-time PEMFC EIS	TAB-converter voltage perturbation with FFT-based impedance extraction; 1 Hz–5 kHz	Converter-based real-time EIS approach; useful contrast with the present DDS/VCCS excitation and analog MRPDD extraction strategy
[17]	Metrological battery EIS	Calibrated EIS with uncertainty propagation; 100 mHz–5 kHz	Strong reference for low-impedance error sources and correction needs; not an embedded architecture
[18]	Embedded bioimpedance spectroscopy	Comparison of GPD/AD8302, IQ, and FFT methods; 1 kHz–1 MHz	Methodological reference for selecting GPD/MRPDD-type extraction; different application domain
[19]	Compact Li-ion EIS instrumentation	Compact low-power battery EIS unit; analog vector processing; 0.1 Hz–15 kHz	Strong compact battery-EIS reference; closer in objective, but based on a different front-end philosophy
[12]	Portable bioimpedance spectroscopy	MRPDD with AD8302 and reference resistor; 20 kHz–1 MHz	Foundational MRPDD reference; much higher impedance and frequency range than Li-ion battery EIS

**Table 2:** Summary of the performance of the proposed EIS sensor system.

Parameter	Specification	Details
Battery range	0.9 Hz–1 kHz	Set by error and phase-readout limits.
Validated magnitude range	100 Hz–10 kHz	Error below 5% versus HP 4263B LCR.
Impedance range	Few-milliohm to sub-ohm	For 18650 cells and low-impedance *RC* models.
Excitation	Single-sine	Sweep generated by the AD9837 DDS.
Measurement method	Analog MRPDD + generalized correction	Correction of $Z_{S}$ and $Z_{par}$.
Tested cell	Samsung ICR18650-26J, 2.6 Ah	Measured at 0.15 C and 27–32 °C.
Current source	LM13700 OTA + IRF3808 NMOS	High output impedance and stable operation.
Differential acquisition	Two AD8429 channels + programmable gain	Acquisition of $v_{Z}$ and $v_{S}$.
Magnitude/phase readout	AD8302 + oscilloscope support	Oscilloscope used for low-frequency phase.
Control and display	Seeeduino XIAO + 0.96” OLED	Computes $Z_{X}$, displays data, controls sweep.

## Data Availability

The original contributions presented in this study are included in the article. The PCB design and fabrication files of the proposed prototype, including the Gerber files and the 3D STEP model of the board, are openly available in a public GitHub repository at: https://github.com/JuanMariaNogales/Generalized-MRPDD-EIS-PCB (accessed on 19 May 2026).
